# Bias, Accuracy, and Impact of Indirect Genetic Effects in Infectious Diseases

**DOI:** 10.3389/fgene.2012.00215

**Published:** 2012-10-22

**Authors:** Debby Lipschutz-Powell, J. A. Woolliams, P. Bijma, R. Pong-Wong, M. L. Bermingham, A. B. Doeschl-Wilson

**Affiliations:** ^1^The Roslin Institute and Royal (Dick) School of Veterinary Studies, Genetics and Genomics, University of EdinburghMidlothian, UK; ^2^Animal Breeding and Genomics Centre, Wageningen UniversityWageningen, Netherlands

**Keywords:** associative, indirect genetic, social interaction, infectious disease, breeding, infectivity, super spreaders, binary

## Abstract

Selection for improved host response to infectious disease offers a desirable alternative to chemical treatment but has proven difficult in practice, due to low heritability estimates of disease traits. Disease data from field studies is often binary, indicating whether an individual has become infected or not following exposure to an infectious disease. Numerous studies have shown that from this data one can infer genetic variation in individuals’ underlying susceptibility. In a previous study, we showed that with an indirect genetic effect (IGE) model it is possible to capture some genetic variation in infectivity, if present, as well as in susceptibility. Infectivity is the propensity of transmitting infection upon contact with a susceptible individual. It is an important factor determining the severity of an epidemic. However, there are severe shortcomings with the Standard IGE models as they do not accommodate the dynamic nature of disease data. Here we adjust the Standard IGE model to (1) make expression of infectivity dependent on the individuals’ disease status (Case Model) and (2) to include timing of infection (Case-ordered Model). The models are evaluated by comparing impact of selection, bias, and accuracy of each model using simulated binary disease data. These were generated for populations with known variation in susceptibility and infectivity thus allowing comparisons between estimated and true breeding values. Overall the Case Model provided better estimates for host genetic susceptibility and infectivity compared to the Standard Model in terms of bias, impact, and accuracy. Furthermore, these estimates were strongly influenced by epidemiological characteristics. However, surprisingly, the Case-Ordered model performed considerably worse than the Standard and the Case Models, pointing toward limitations in incorporating disease dynamics into conventional variance component estimation methodology and software used in animal breeding.

## Introduction

Infectious diseases in livestock constitute a major threat to the sustainability of livestock production. Reducing disease prevalence through selection for host resistance offers a desirable alternative to chemical treatment. Particularly since recent advances in high throughput genomic information has led to new opportunities for dissecting genetic variation and to accelerate genetic improvement. However, a major barrier to closing the genotype–phenotype gap is uncovering the genetic variance underlying disease phenotypes. To do so, genetic analyses require large sample sizes and hence disease phenotypes often need to be obtained from field data. Bishop and Woolliams (2010) have demonstrated that shortcomings of current estimation methods for field data which fail to take epidemiological considerations into account cause seemingly low heritability estimates for disease traits in domestic livestock. For example, in a previous study (Lipschutz-Powell et al., 2012a), it was demonstrated that conventional statistical models used for variance component estimation cannot capture genetic variation in host infectivity, when present in disease data, as they consider exposure as an environmental factor.

Host infectivity is the propensity of an infected individual to infect its group mates. The lack of attention to host variation in infectivity in genetic studies stands in stark contrast to the well-recognized important role of host infectivity in epidemiology. There is abundant evidence that heterogeneity in infectivity is ubiquitous, super-shedders being an extreme example, and can profoundly impact upon disease prevalence in the population (Woolhouse et al., 1997; Lloyd-Smith et al., 2005; Yates et al., 2006; Doeschl-Wilson et al., 2011). However, it is not known to what extent infectivity is genetically controlled as it is difficult to measure directly. Evolutionary arguments would however suggest that there should be a significant amount of genetic variation in infectivity, because infectivity is not a component of an individual’s fitness. Accumulation of genetic variation will, therefore, not be prevented by natural selection (Denison et al., 2003).

Disease data from field studies is often binary, indicating whether an individual became infected or not following exposure to infectious pathogens. Numerous studies have demonstrated that from this data one can infer genetic variation in individuals’ underlying susceptibility to that disease (e.g., Houston et al., 2010). However, standard models do not lend themselves to estimating genetic variation in infectivity as the effect of infectivity is observed in a different individual than the one expressing it (Lipschutz-Powell et al., 2012a). The theory of indirect genetic effects (IGE), also known as associative or social genetic effects, provides an appropriate framework to account for genetic variation in infectivity as it investigates heritable effects of an individual on the trait value of another individual (Griffing, 1967). In this context, host infectivity can be regarded as an indirect effect to disease status.

Lipschutz-Powell et al. (2012a) was the first study to demonstrate that genetic variation in host infectivity can be captured to some extent from binary disease data using an IGE model. However the results of that study suggest that there are severe shortcomings in using the standard IGE model, to estimate genetic variance in infectivity. For example the standard IGE model assumes that all individuals express the indirect effect (infectivity) at all times and that all individuals are affected by the indirect effect. However, only individuals who are infected can express infectivity. Furthermore, the infectivity of an infected individual will affect the disease status of susceptible group members only. Moreover, the number of both susceptible and infected individuals will change over time. In this way, the gross underestimation of genetic variance in infectivity observed in Lipschutz-Powell et al. (2012a) may have occurred because the IGE was attributed to all individuals in a group when in reality it was expressed by and affecting only a subset of group members. Our hypothesis is, therefore, that an IGE model, when used to analyze binary disease data, may be improved by accounting for disease dynamics.

Here we explore the implementation of dynamic properties within the remit of a conventional quantitative genetics mixed model framework and software (ASReml, Gilmour et al., 2006). To do so we specify which individuals contribute to an effect using the incidence matrix. In this study, two adjustments to the standard statistical IGE model were made. The first model, denoted the *Case Model*, accounted for the fact that only infected individuals can express infectivity. The second model, denoted the *Case-Ordered Model*, also accounted for the fact that infected individuals can only affect individuals who did not become infected before them. To evaluate these adjusted statistical models, we modeled disease progression in populations with genetic variation in host infectivity and susceptibility and estimated the genetic (co)variances in the simulated binary disease data with each model. The populations were simulated with varying epidemiological characteristics in order to assess their impact on our estimates. Finally, we evaluated the bias, accuracy, and impact of the estimates obtained with each model and compared them to those obtained with the Standard (unadjusted) IGE model.

## Materials and Methods

### The statistical models

#### Standard IGE model

The Standard IGE model has been described by Muir (2005). Thus for disease phenotype *y* (e.g., infected or not) observed in individual *j* living in group *h* of size *n* with group mates *m*

$$\begin{array}{rcll} & y_{jh}\sim \text{mean}+{\left(\text{direct}\;\text{effect}\right)}_{j}+\overset{n-1}{\underset{m=1}{\sum }}{\left(\text{indirect}\;\text{effect}\right)}_{mh} & & \\ & +{\left(\text{group}\;\text{effect}\right)}_{h}+e_{jh}. & & \text{(1)} \end{array}$$

Bijma et al. (2007) demonstrated the integration of this model into the quantitative genetics mixed models framework to obtain estimates for genetic variances and co-variances of direct and IGEs. In the context of infectious disease, the direct effect relates to host susceptibility and the indirect effect to host infectivity. In the statistical analysis, the direct, indirect, and group effects were all fitted as random effects. According to Eq. 1, for the Standard IGE model it is assumed that the direct and indirect effects are expressed by all individuals (i.e., expression does not depend on the disease status of an individual or that of its group mates).

#### Case IGE model

For the Case IGE model, model (1) was expanded to account for the fact that only infected individuals can express the indirect effect

$$\begin{array}{llll} & y_{jh}\sim \text{mean}+{\left(\text{direct}\;\text{effect}\right)}_{j} & & \\ & +\overset{n-1}{\underset{m=1}{\sum }}X_{mh}{\left(\text{indirect}\;\text{effect}\right)}_{mh}+{\left(\text{group}\;\text{effect}\right)}_{h}+e_{jh}. & & \text{(2)} \end{array}$$

Where the indicator trait *X_mh_* is equal to one if *m* became infected during the recording period and zero otherwise. In this way the number of individuals contributing to the IGE $\left(\sum_{m=1}^{n-1}X_{mh}\right)$ will be equal to the number of group mates that have become infected during the observation period, representing the group’s total exposure. The number of infected individuals will vary between groups not only for genetic reasons, but also due to environmental factors or chance. This creates a non-genetic covariance among group mates, which is accounted for by the random group effect. It is assumed that the population is naïve at the start of the recording period and that all individuals express susceptibility, although to varying extent (see Simulated Populations).

#### Case-ordered IGE model

For the Case-ordered IGE model the Case model was expanded to include the order of infection of individuals, thus accounting for the fact that an infected individual *m* can only impact on group members that did not become infected prior to its own infection

$$\begin{array}{llll} & y_{jh}\sim \text{mean}+\psi \overset{n-1}{\underset{m=1}{\sum }}X_{m_{j}h}+{\left(\text{direct}\;\text{effect}\right)}_{j} & & \\ & +\overset{n-1}{\underset{m=1}{\sum }}X_{m_{j}h}{\left(\text{indirect}\;\text{effect}\right)}_{mh} & & \\ & +{\left(\text{group}\;\text{effect}\right)}_{h}+e_{jh}. & & \text{(3)} \end{array}$$

The indicator trait $X_{m_{j}h}$ is equal to one if the group mate *m* became infected before individual *j*. The number of individuals contributing to the IGE $\left(\sum_{m=1}^{\text{n - 1}}X_{m_{j}h}\right)$, i.e., the exposure faced by individual *j*, will now vary between group mates and has *n* − 1 possible levels. To account for differences in exposure between group mates, the effect of individual exposure $\left(\psi \sum_{m=1}^{n-1}X_{m_{j}h}\right)$ was fitted as a separate fixed effect.

#### Variance structure

It was assumed that group mates are unrelated and all effects are independent of the residuals. The Standard IGE model can be written in the form of the Case IGE (2) with a constant indicator trait *X_mh_* = 1which has an expectation of one and zero variance. Assuming that all effects are independent of the residuals and given that *E*(*X*^2^) = *E*(*X*) as *X* is binary, the phenotypic variance for all three models can be partitioned, for a given level of individual exposure, as follows:

(4) $$\sigma_{y}^{2}=\sigma_{d}^{2}+\left(n-1\right)E\left(X\right)\sigma_{i}^{2}+\sigma_{\text{group}}^{2}+\sigma_{e}^{2}.$$

Where *d* stands for the direct and *i* for the indirect effect. Thus the phenotypic variance for all three models differs only in the components pertaining to the indicator trait *X*, i.e., *E*(*X*) which is the proportion of group mates expected to contribute to the IGE.

For the model fitting it was assumed that the vector of observed traits **y** follows a multi-variate normal distribution with means given by the fixed effects and the following variance structure:

(5) $$\text{Var(y)}={\text{Z}}_{a}{\text{G}}_{a}{\text{Z}}_{a}^{\prime }+{\text{Z}}_{e}{\text{G}}_{e}{\text{Z}}_{e}^{\prime }+\text{R}$$

Where **R** and **G*_e_*** are diagonal matrices with the residual and group variances, respectively, on the diagonal. **G***_a_* is the genetic (co)variance matrix and is given by the Kronecker product of a two by two variance-covariance matrix of direct and indirect effects and the relationship (**A**) matrix. **Z***_a_* is an incidence matrix linking individuals to their direct and indirect effects and **Z*_e_*** an incidence matrix linking individuals to their group. Thus at each individual level, term four of Eq. 4 is given on the diagonal of **R** and term three on the diagonal of **G*_e_***. As individuals are expected to be unrelated within groups, the direct-indirect covariance should not contribute to the phenotypic variance. The incidence matrix **Z***_a_* links one direct effects variance from **G***_a_* to the phenotypic variance, i.e., term one of Eq. 4, and $\sum_{m=1}^{n-1}X_{m_{j}h}$ indirect effects variances. Hence Eq. 4 is the expectation of the phenotypic variances given by Eq. 5.

### Simulated data

To evaluate the three models, simulated binary disease data were generated. For this purpose, epidemics were simulated with genetic variation in host susceptibility and infectivity following known distributions.

#### The epidemiological model

An epidemic was simulated in a population consisting of many groups (see Simulated Populations below). The simulation describes disease progression in each group and provides as output the disease status of each individual at given time points. These provided the binary disease records used for fitting the statistical models described in Section “The Statistical Models.” To avoid overburdening the results with unnecessary complexity we chose a simple compartmental stochastic susceptible-infected-recovered (SIR) model as detailed in Lipschutz-Powell et al. (2012a). In an SIR-model, individuals can be in one of three disease states, being susceptible (*S*), infected (*I*), or recovered (*R*). Individuals move through states in the order *S* → *I* → *R*. Initially, all individuals are in the *S*-state. Upon infection, a susceptible individual moves from the *S*-state to the *I*-state. Upon recovery, an infected individual moves to the *R*-state. The average rate of transition between the epidemiological compartments *S*, and *I* is determined by the transmission parameter β, whereas the average rate of transition between the compartments *I* and *R* is determined by the recovery rate γ. It was assumed that infected individuals become immediately infectious.

Genetic variation in host susceptibility and infectivity was incorporated into the model by assigning for each individual *j* its own level of susceptibility *g_j_* and infectivity *f_j_*. Hence, there is no longer a fixed transmission parameter β for the entire population, but the rate of transmission from individual *k* to individual *j* is given by the pair-wise transmission parameter β*_jk_*, which depends on the infectivity of *k* and the susceptibility of *j*. In order to reduce unnecessary noise it was assumed that variation in susceptibility and infectivity was fully genetic. However, the outcome, i.e., whether an individual became infected or not, was assumed to be a stochastic event and will therefore contain both a genetic and a random non-genetic component. The pair-wise transmission parameter β*_jk_* was derived from first principles in Lipschutz-Powell et al. (2012a) and defined as

(6) $$\beta_{jk}=-\ln\left(1-X_{g,j\;}g_{j}X_{f,k}f_{k}\right).$$

Thus β*_jk_* is a function of the product of the susceptibility *g* of individual *j* and the infectivity *f* of individual *k*. To reflect whether susceptibility is expressed by individual *j*, it is scaled by *X_g,j_* which equals one if *j* is susceptible and zero otherwise. Similarly, infectivity is scaled by *X_f,k_* which equals one if *k* is infected and zero otherwise. In this way, transmission between individuals *j* and *k* can only occur if *j* is susceptible and *k* infectious. For simplicity no variation in individual speed of recovery γ*_k_* was assumed. Hence, individual speeds of recovery γ_k_ were assumed to be equal to a constant γ if the individual was infected and zero otherwise.

The epidemic was simulated as a stochastic Poisson process as detailed in Lipschutz-Powell et al. (2012a) which starts by infecting one randomly chosen individual in each group and describes disease progression in the population through a series of independent infection and recovery events. No transmission was assumed between groups.

#### Simulated populations

Following Lipschutz-Powell et al. (2012a) the simulated populations consisted of *N* = 100,000 individuals with a paternal half-sib structure and no full sibs. All parents were assumed to be unrelated. The half-sib family size was 100 individuals. Each population was divided into 10,000 groups of size *n* = 10 chosen at random without reference to pedigree. Since each population was divided into 10,000 groups giving rise to 10,000 independent epidemics, each simulation was replicated only 10 times. The simulation was run for populations with variation introduced in both susceptibility and infectivity. Note that none of the IGE models presented in this paper take individuals’ recovery into account. Therefore, to assess the impact of recovery speed, on the outcome of the subsequent analyses, the simulations were run for populations with constant speed of recovery γ = 0.1, 0.01, or 0.001. These populations will be referred to as having a high, medium, or low recovery rate, respectively.

Similarly to Lipschutz-Powell et al. (2012a) breeding values (BVs) for susceptibility and infectivity were assumed to be distributed according to the right-skewed gamma distribution γ(*a*, þ). This distribution was chosen because the distribution of infectivity is often right-skewed (Lloyd-Smith et al., 2005). Moreover, skewed distributions allow for larger variation when the distribution is confined to positive values. The same parameters were chosen for both susceptibility and infectivity in order to allow for direct comparisons. Specifically, the parameters were taken as *a* = 1.14 and þeta = 0.18, such that the mean *a*þeta = 0.21 the variance *a*þ^2^ = 0.037 and the distribution is right-skewed with skewness $2/\sqrt{a}$ = 1.87. For details on how the BVs of the offspring generation were constructed from the parental generation, refer to Lipschutz-Powell et al. (2012a).

A recent study showed molecular evidence for a positive correlation between susceptibility and infectivity as the known immunosuppressant stress hormone norepinephrine was shown to cause increase shedding of *Salmonella* (Pullinger et al., 2010). In order to examine the impact of such covariation between susceptibility and infectivity, the correlation between both parameters were either set to 0 or 0.35. If no correlation was assumed, the BVs were assigned to individuals as detailed in Lipschutz-Powell et al. (2012a). Non-zero correlations were generated by assigning parental BVs using the gamma trivariate reduction algorithm as specified in Schmeiser and Lal (1982).

### Validation of the statistical models

#### Estimating genetic parameters from simulated data

Genetic parameters and BVs associated with host susceptibility (direct effect) and infectivity (indirect effect) were estimated with the three statistical models presented in Section “The Statistical Models.” The phenotypes used for this purpose were binary records describing the disease state of the simulated individuals during a given recording time. The latter was chosen such that the mean number of infected individuals per group was approximately *n*/2 in all populations. The binary disease trait, denoted here as “disease presence,” was one if an individual had become infected during the recording time and zero otherwise.

The Case-Ordered model (3) not only required information on the disease state of individuals but also on the order of infection within each group. Infection occurs over a continuous time scale. However, in practice data is often recorded at discrete sampling times. Knowledge of the exact order would in practice be equivalent to dividing the recording period into an infinite number of sampling times. Here the Case-Ordered model was simplified so that the recording time was split into two sampling times. Thus the records used in the analyses were the disease presence of individuals recorded at the two sampling times. The length of each period was taken such that approximately half of the individuals that had become infected by the end of the recording time, would have become infected during the first period and the other half in the second period. The reasoning behind this choice in sampling times is outlined in the discussion. Thus the indicator trait $X_{m_{j}h}$ for individual *j* with group mate *m* in Eq. 3 was equal to one only if group mate *m* had become infected and *j* was still susceptible at the start of the recording period in which *m* became infected.

The phenotypes of randomly chosen individuals initiating the epidemic in each group were removed prior to analysis. The genetic information of these individuals was however included in the analysis. Due to difficulties with convergence (see Discussion) the direct-indirect covariance was fixed prior to analysis to zero when susceptibility and infectivity were independent and to 0.014, in correspondence with the simulated correlation, otherwise. All genetic analyses were carried out using ASReml (Gilmour et al., 2006).

#### Validation criteria

##### Expected variance

In the simulations, genetic variation was introduced in the underlying parameters infectivity and susceptibility. The analysis of simulated data, however, was performed at the level of observed disease status (0, 1). To judge the results of the data analysis, i.e., to compare the estimated values to their expected values, it is necessary to transform susceptibility and infectivity to the observed binary scale. Following Dempster and Lerner (1950) we assumed a linear relationship between the susceptibility and the observed binary phenotype of an individual and between the infectivity of an individual and the binary phenotypes of its group mates. Specifically, the effect of susceptibility was obtained by regressing the individuals’ phenotypes *y* on their susceptibility *g* (*y* ∼ *b*_1_*g*). To obtain the relationship between infectivity and the disease status of an individual’s group mates, the phenotypes *y* of individuals *j* were regressed on the infectivity *f* of a randomly chosen infected group mate *k* of *j* (*y_jh_* ∼ *b*_2_*f_kh_*). This approximation was used as there are many groups and relatively few group mates. The corresponding regression coefficients *b*_1_ and *b*_2_ were estimated using the statistical package R (Ihaka and Gentleman, 1996) using the known BVs and phenotypes from the simulations. Similarly to the genetic analysis, the phenotypes of the individuals which were randomly chosen to initiate the epidemic were discarded. In this way, the true direct BV of individual *j* (BV*d_j_*) corresponds to *b*_1_*g_j_* and its indirect BV (BV*i_j_*) to *b*_2_*f_j_*. The expected (co)variances for the direct and indirect effects are then given by the (co)variances of their corresponding true BVs. The expected phenotypic variance was estimated as $\bar{p}(1-\bar{p}),\bar{p}$ denoting mean prevalence.

##### Bias and accuracy

Estimates of bias for both the direct and indirect effects were obtained by regressing the true BVs for direct and indirect effects (as derived above) on the corresponding estimated breeding values (EBVs) obtained from each model.

Accuracy was estimated as the correlations between the true BVs for susceptibility and infectivity with the corresponding direct and indirect effect EBVs. Note that transformation of susceptibility/infectivity BVs to binary scale BVs was not necessary for calculating correlations under the assumption of a linear relationship between the underlying and observed scales.

##### Impact of selection

In order to estimate the impact of the three models on response to selection the population true mean susceptibility/infectivity was compared to the true mean susceptibility/infectivity after selection of 10% of the individuals with the lowest EBVs obtained from each model. For all three models, selection was carried out based upon the EBVs for direct effect (EBV*_d_*) and indirect genetic effect (EBV*_i_*) separately as well as for the index *I*_x_ = EBVd + *x*EBVi with *x* taken as the product of the expected number of individuals contributing to the IGE and the expected total group exposure rounded to the nearest integer. Specifically, *x* = 4, 2, and 1 for the Standard, Case, and Case-ordered model, respectively. These weights were chosen to take into account the number of individuals contributing to the indirect effect as well as the level of exposure. Moreover, they provided the greatest impact when tested on the population with zero correlation between susceptibility and infectivity and recovery rate 0.01.

To quantify response to selection in terms of risk and severity of the epidemic, the basic reproduction number *R*_0_ was estimated for the whole population and for each selected subpopulation using the true values of susceptibility and infectivity from the simulation. *R*_0_ is the mean number of secondary infections an infected individual will cause in its lifetime in an otherwise naïve population, and is commonly used as a measure of disease risk and severity in epidemiology (Anderson and May, 2006). By definition an epidemic will die out if *R*_0_ < 1. Following a SIR-model for a closed population, *R*_0_ = β*S*_0_/γ, *S*_0_ = (*n* − 1) being the initial number of susceptible individuals in a group (Keeling and Rohani, 2008). Incorporating Eq. 6 and taking a Taylor series expansion we obtain as zero order approximation

(7) $$R_{0}\approx \frac{\left(-\text{ln}\;\left(1-ḡ\bar{f}\right)S_{0}\right)}{\gamma }$$

## Results

All results presented in Sections “Variance Estimates,” “Bias and Accuracy,” and “Impact of Selection” refer to the populations with a zero correlation between infectivity and susceptibility.

### Variance estimates

Table 1 shows the variance estimates obtained for each population, from all three models, along with the expected variances. Overall the variance estimates obtained with the Case model are in best agreement with the expected variances. This is particularly true for the populations with a medium to slow recovery rate. Whilst the Standard model vastly underestimates the IGEs variance, the Case-ordered model provides vastly inflated estimates. Moreover, in contrast to the Standard and Case Models, the Case-ordered model also grossly underestimates the direct effects variance. From this it is clear that the Case-ordered model is inadequate. Therefore further results for the Case-ordered model will not be shown as they merely confirmed the overall poor performance of this model. Potential explanations and alternative suggestions are outlined in the discussion section of this paper.

**Table 1:** **Genetic variance estimates**.

Effect	Recovery rate γ	Model			
		Expected	Standard	Case	Case-ordered
Direct	0.1	20.61	20.28	23.84	9.65
	0.01	33.79	35.07	37.28	11.45
	0.001	34.30	35.26	36.73	9.03
Indirect	0.1	16.70	0.33	6.72	38.80
	0.01	8.25	0.72	6.32	86.47
	0.001	5.52	0.70	7.08	108.45

*Expected and estimated genetic variance in the direct and indirect effect in populations with a high, medium, and low recovery rate. Variance components were estimated with the Standard, Case, and Case-ordered models. All variance components have been scaled by 10^3^*.

The indirect effects variance estimate obtained with the Case model deviates most from the expected variance in the population with a high recovery rate (γ = 0.1), i.e., when infected individuals are most likely to recover during the epidemic. This is not surprising, as the Case Model does not take the time period of infection into account. Thus, individuals that recover early would be assumed to contribute infectivity during the entire recording period. It is also noteworthy that the direct effects variance (both expected and estimated) increases and the indirect variance decreases with decreasing recovery rate. This demonstrates that the relative contributions of both effects to the overall variance strongly depend on epidemiological characteristics.

### Bias and accuracy

**Figure 1:**
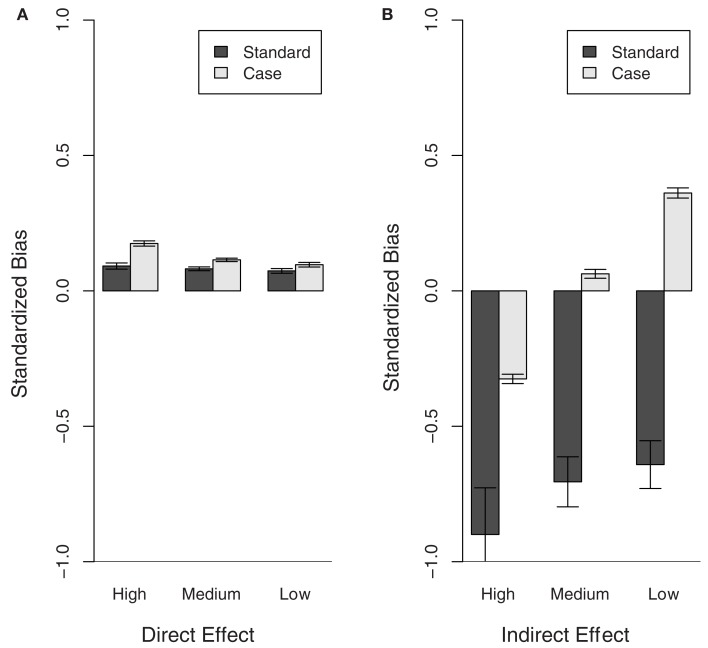
**Bias of direct and indirect effect BV estimates for populations with different recovery rates (High, Medium, and Low)**. The bias estimates (regression coefficient of the true BVs on the EBVs), obtained for the Case and Standard model, were standardized to 1-bias if bias <1 and 1/bias-1 if bias >1, in order to show over and under estimation of the effects at the same scale. Thus values >0 show over-estimation and values <0 underestimation of the breeding values. **(A)** Direct effect, **(B)** indirect effect.

Figure 1 shows the standardized bias estimates obtained for each population from the Standard and Case models. These results confirm the conclusions from the comparison between estimated and expected variance components (see Table 1). Specifically, the BV estimates obtained for the direct effect show little bias with either model. However, the Standard model grossly underestimates the indirect effect BV. It is noteworthy that, whilst the estimates obtained with the Case model show less bias overall, both the degree and direction of the standardized bias estimates depended on the recovery rate. Specifically, the standardized bias estimates for the indirect effect obtained with either model show an upward trend as the recovery decreases. This is in line with the results of Section “Variance Estimates” showing that the expected variance in the indirect effect decreases with the recovery rate whereas the estimated variance in the indirect effect obtained with either the Standard or Case model remain more or less constant. This suggests that epidemiological characteristics affect the bias of indirect effect estimates and further improvements may be possible if these are properly accounted for.

**Figure 2:**
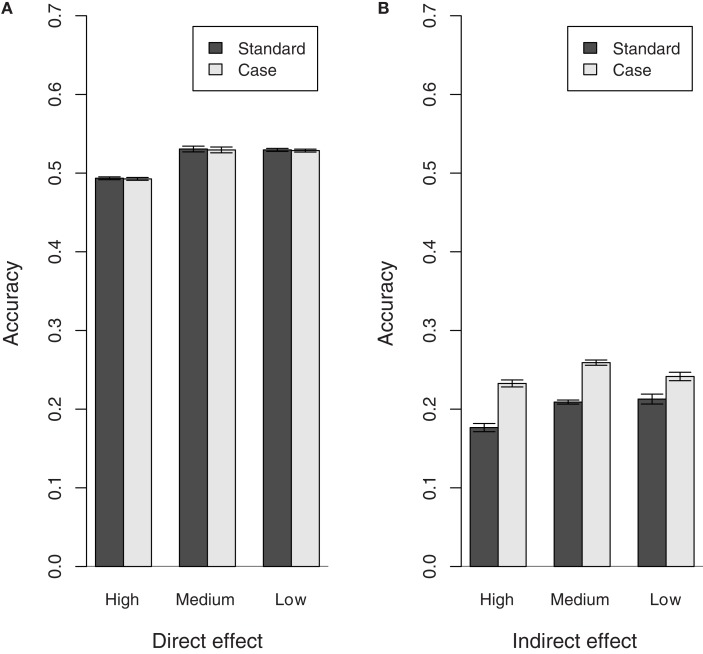
**Accuracy of direct and indirect effect BV estimates for populations with different recovery rates (High, Medium, and Low)**. **(A)** Direct effect, **(B)** indirect effect.

Figure 2 shows the accuracy estimates obtained for each population, for the Standard and Case models. The accuracy of the direct effect BV obtained for the Case model is similar to that obtained for the Standard model in all populations. However, the indirect effect BV estimates obtained with the Case model have a greater accuracy compared with those obtained with the Standard model. Also, there is a slight increase in the accuracy of the direct effect BV estimates obtained with the Standard and the Case model as the recovery rate decreases. This coincides with the increase in expected variance of the direct effect. It may also be due to the fact that for both the Standard and the Case model it is assumed that individuals express infectivity throughout the observation period. This assumption becomes more valid as individuals become less likely to recover, i.e., as the recovery rate decreases.

Note that the accuracy estimates obtained for the direct effect BVs with the Standard and Case model are reasonable given the half-sib structure of the population. The accuracy estimates obtained for the indirect effect BVs, on the other hand, are much lower, thus indicating that there is still further scope for improvement.

### Impact of selection

Table 2 shows the true mean susceptibility and infectivity values and the basic reproductive ratio *R*_0_ (scaled by γ) after selection using the EBVs obtained with the Standard and Case model from each population. Overall, selection using an index of direct and indirect EBVs obtained with the Case model shows slightly more reduction in risk and severity of an epidemic as measured by *R*_0_. However, the difference between the use of an index and the direct effect EBVs alone is small. This makes sense given the low accuracy estimates obtained for the indirect effect EBVs. The benefits of the Case Model over the Standard Model are mainly caused by the improved estimates for the indirect effects EBVs. Whilst selection on the direct EBVs from the Case model made little to no difference on true mean susceptibility compared with selecting on the direct EBVs from the Standard model, selection on the indirect EBVs from the Case modeled to greater reduction of true mean infectivity.

**Table 2:** **Selection impact on true susceptibility, infectivity, and risk and severity of an epidemic**.

Recovery rate γ	Model	Selected on	Mean susceptibility	Mean infectivity	*R*_0_ · γ
		No selection	0.21	0.21	0.41
0.1	Standard	EBV*_d_*	0.07	0.22	0.15
		EBV*_i_*	0.24	0.15	0.34
		EBV*_x_*	0.08	0.21	0.15
	Case	EBV*_d_*	0.08	0.22	0.15
		EBV*_i_*	0.18	0.13	0.22
		EBV*_x_*	0.08	0.19	0.14
0.01	Standard	EBV*_d_*	0.08	0.21	0.15
		EBV*_i_*	0.24	0.15	0.33
		EBV*_x_*	0.08	0.20	0.15
	Case	EBV*_d_*	0.08	0.21	0.15
		EBV*_i_*	0.22	0.13	0.26
		EBV*_x_*	0.08	0.19	0.14
0.001	Standard	EBV*_d_*	0.08	0.21	0.15
		EBV*_i_*	0.23	0.15	0.32 ± 0.01
		EBV*_x_*	0.09	0.19	0.15
	Case	EBV*_d_*	0.08	0.21	0.15
		EBV*_i_*	0.24	0.14	0.30
		EBV*_x_*	0.08	0.19	0.14

*For all three models, selection was carried out based upon the EBVs for direct effect (EBV*_d_*) and indirect genetic effect (EBV*_i_*) separately as well as for the index *I_x_* = EBV*_d_* + *x*EBV*_i_*, with *x* taken as the product of the expected number of individuals contributing to the IGE and the expected total group exposure rounded to the nearest integer. Specifically, *x *= 4, 2, and 1 for the Standard, Case, and Case-ordered model, respectively. Standard errors all <5 · 10^−3^ unless indicated*.

It is noteworthy that the mean susceptibility increases, when selecting on the indirect effect EBVs from all analyses except for the Case model with a high recovery rate. This general increase in mean susceptibility can be explained by the fact that only infected individuals can express infectivity. Thus individuals with a low susceptibility are less likely to express infectivity. It is therefore less likely that the EBV for infectivity of these individual would be on the extreme (selected) ends of the distribution. However, there is a slight decrease, rather than increase, in mean susceptibility, when selecting on the indirect effect EBVs obtained with the Case model from the population with a high recovery rate. This may be explained by the following. As seen in Section “Variance Estimates,” the effect of infectivity increases as the recovery rate increases probably due to an increase in the importance of being infected early (high susceptibility). Hence, individuals with a high susceptibility are more likely to be assigned a high infectivity EBV as the recovery rate increases. This is in line with the fact that the mean susceptibility, when selecting on indirect effect EBVs obtained with the Case model, increases as the recovery rate decreases.

### Effect of dependence between susceptibility and infectivity

**Figure 3:**
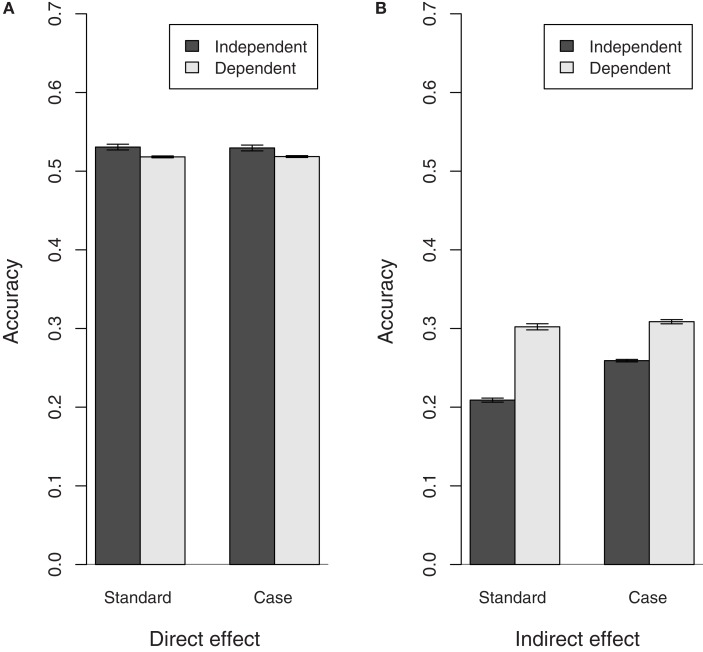
**Accuracy of direct and indirect effect estimates in populations with(out) dependence between susceptibility and infectivity**. Results shown for populations with a medium recovery rate, similar results were obtained for populations with different recovery rates. The correlation between susceptibility and infectivity is 0 in the independent population and 0.35 in the dependent population. **(A)** Direct effect, **(B)** indirect effect.

Having a positive correlation between susceptibility and infectivity had little to no impact on the bias of all estimates (results not shown) and the accuracy of the direct effect variance estimates. The accuracy of all indirect effect estimates, however, increased when there was a positive correlation between susceptibility and infectivity (Figure 3). This increase in the accuracy of the indirect effect EBVs may be due to the fact that the accuracy of EBVs obtained by Best Linear Unbiased Prediction inherently improves when they are positively correlated (Falconer and MacKay, 1996). Moreover, it may also be due to the fact that whether infectivity is expressed or not depends on susceptibility, even if infectivity and susceptibility themselves are independent. In that way, the indirect effect EBVs will partly depend on susceptibility and hence they will be more accurate if there truly is a positive correlation between infectivity and susceptibility.

Finally, the impact of selection on true mean susceptibility, infectivity, and *R*_0_ is compared between populations with and without dependency between susceptibility and infectivity, and a medium recovery rate, in Table 3. As may be expected, the greatest impact of selection on *R*_0_ was obtained in the population with a positive correlation between susceptibility and infectivity. A similar improvement was observed in populations with other recovery rates (results not shown).

**Table 3:** **Selection impact in a population with a positive correlation between susceptibility and infectivity**.

	Correlation	Mean susceptibility		Mean infectivity		*R*_0_ · γ	
		0	0.35	0	0.35	0	0.35
	No selection	0.21	0.21	0.21	0.21	0.41	0.41
Standard	EBV*_d_*	0.08	0.08	0.21	0.14	0.15	0.10
	EBV*_i_*	0.24	0.10	0.15	0.13	0.33	0.12
	EBV*_x_*	0.08	0.08	0.20	0.13	0.15	0.10
Case	EBV*_d_*	0.08	0.08	0.21	0.14	0.15	0.10
	EBV*_i_*	0.22	0.15	0.13	0.13	0.26	0.17
	EBV*_x_*	0.08	0.08	0.19	0.12	0.14	0.09

*For populations with a medium recovery rate γ = 0.01. Standard errors all <5 · 10^−3^*.

Overall, the performance of the Standard model was closer to that of the Case model when there was a positive correlation between susceptibility and infectivity. Note that in order to achieve convergence the covariance estimate was fixed in all analyses. Varying the value at which the covariance is fixed slightly affected the bias estimates but not the accuracy nor any of the previous observations.

## Discussion

We have previously shown (Lipschutz-Powell et al., 2012a) that IGE models developed for production traits provide a promising tool for estimating genetic variation underlying binary disease data. However, standard IGE models did not fully capture genetic variation in infectivity. The hypothesis of this study was that extending an IGE model to allow for disease dynamics ought to improve its ability to estimate genetic variation in susceptibility and infectivity from binary disease data. Here we explored the extent to which it is possible to do so, within the remit of the conventional mixed model framework and software. In these conditions it is possible to specify the individuals contributing to the indirect effect using the incidence matrix. The effect of including disease dynamics, in this way, was assessed by comparing the accuracy, bias, and selection impact of two adjusted IGE models, with the Standard IGE model, using simulated data. In the first adjusted IGE model, the Case model, it was assumed that only infected individuals have an indirect effect on their group mates. In the second adjusted IGE model, the Case-ordered model, it was assumed that infected individuals only have an indirect effect on susceptible group mates. Our results show that taking the disease status of individuals into account, by using the Case model, considerably improved the bias, and showed some improvement in accuracy and impact of genetic infectivity estimates from binary disease data compared to the Standard model. However, although heuristically one would assume that the Case-ordered model would provide the best estimates, as it takes most of the disease dynamics into account, in fact it provides the worst.

The poor performance of the Case-ordered model reveals that the straight-forward approach of incorporating information about disease dynamics in the form of incidence matrices into a linear mixed model has severe limitations. The problem with using incidence matrices containing information about individuals contributing to the indirect effect as explanatory variables (i.e., on the right-hand-side of the statistical model) is that they use information obtained from the very observations they try to predict. Thus, the indicator traits, like the observations, are partly determined by the BVs we are trying to estimate. Note that this was also true for the Case model, but to a much lesser extent as the indicator trait corresponds to the disease state of another individual in that model. However, the indicator trait in the Case-ordered model is a property of the susceptibility of two individuals, potentially rendering the numerical relationships too complex for the estimation software used. In fact, a much simplified approximation had to be used for the Case-ordered model in order to achieve convergence. Indeed, the number of observation periods had to be reduced to two. Finally, there may be a reduction in statistical power as the number of individuals contributing to the indirect effect decreases. Thus a different approach is needed for implementing information about the order of infection into the statistical models. For example, hierarchical Bayesian models may provide a better framework for incorporating infection order in terms of prior information.

Our results reveal that the contribution of susceptibility and infectivity to an individual’s disease status, as well as the bias and accuracies of the corresponding EBVs obtained with either model, depend on epidemiological characteristics. In particular, the expected direct effect variance will be more important in diseases with a low recovery rate and the expected indirect effect variance will be more important in diseases with a high recovery rate. This may be due to the fact that, when recovery is slow, the exposure will be relatively high as individuals remain infected for longer. Thus not getting infected is more likely the result of a low susceptibility, increasing the relative contribution of variance in susceptibility to the total phenotypic variance. When recovery is fast, on the other hand, having a sufficiently high infectivity in order to spread the infection prior to recovering becomes more important. In accordance with the greater relative contribution of variance in susceptibility, the accuracies of the direct effects EBV are also slightly higher when recovery rate is low. However, the accuracies of the indirect EBVs obtained with the Standard and Case models are decreased in the population with a fast recovery compared with those with a medium or slow recovery. This may be due to the fact that both these models assume a constant expression of infectivity. Hence the assumptions underlying these models are more accurate in populations with a slow recovery. It must also be noted that, including the individuals initiating the epidemic, only about 50% of individuals became infected in all populations. This should be good for estimating the variance in the direct effect but it does mean that approximately 50% of individuals never express infectivity. Further work is therefore required to evaluate the optimal recording time given epidemiological parameters such as recovery rate.

Our results indicate that a positive correlation between susceptibility and infectivity improves the EBVs obtained with all three models in terms of accuracy and impact of selection. The gain in selection impact, when selecting on an index of direct and indirect EBVs, is somewhat expected as selection on either EBV will also be expected to affect response in the other due to dependency. Moreover, it has repeatedly been demonstrated that the covariance between direct and indirect effect is a component of the expected response to selection when using an IGE model (Griffing, 1967). The gain in accuracy of the indirect effect EBVs probably occurs because an individual must be infected, which depends on that individual’s susceptibility, in order to express infectivity. However, these results stem from a specific correlation value and it may be worth investigating whether different correlation values would affect this trend. Similarly the effect of different experimental settings such as grouping related vs. non-related individuals would be worth investigating as they have been demonstrated to strongly affect the scale of parameter estimates (Bijma, 2010).

It must be noted that the model validation partly depended on expected variances and BVs on a binary scale. In this study a simple linear relationship was assumed, following Dempster and Lerner (1950) and Bijma et al. (2007), between the observed binary trait and the underlying genetic parameters. Alternatively, we could have linked the linear mixed model describing the underlying parameters to the binary trait with a non-linear link function using a generalized linear mixed model GLMM. However, the relationship between the underlying genetic parameters and the observed disease status is complex and stochastic. It is therefore unlikely that canonical link functions, such as the probit or logit function, are appropriate in our case. In fact, in Lipschutz-Powell et al. (2012a) we demonstrated that using a GLMM linking the Standard IGE model with our binary disease trait with the logit function provided qualitatively similar results to those obtained without the transformation. Moreover, there was no advantage in using such a transformation as the relationship was not only inappropriate, but it also provided intractable estimates and seemed to increase the interaction bias. We have recently established the appropriate relationship in a further study between the underlying genetic parameters and the observed binary host infectious disease data. By doing so we demonstrated that the probit and logit link functions are inappropriate for the analysis of binary host infectious disease data (Lipschutz-Powell et al., 2012b). Moreover, we demonstrated that the use of a complementary log-log link function or survival analysis is useful when there is variation in susceptibility only but inadequate if there is variation in infectivity (Lipschutz-Powell et al., 2012b). The relationship established by Lipschutz-Powell et al. (2012b) cannot be readily implemented into existing software and is therefore beyond the scope of this study investigating the incorporation of disease dynamics within the framework of a conventional quantitative genetics mixed model and associated software. We therefore decided to use a linear mixed model, which have been shown to provide estimates of genetic parameters of sufficient accuracy to generate selection response (e.g., Vazquez et al., 2009). Other types of models which may be interesting to adapt and develop further in order to estimate genetic parameters associated with host susceptibility and infectivity include cure models (Odegard et al., 2011) and product threshold model (David et al., 2009). Cure models, have the potential to consider expression of infectivity conditional on infection status, whereas product threshold models might better represent the interaction between a susceptible and infectious individual.

In summary, we have shown that epidemiological characteristics and disease dynamics strongly influence estimates of genetic variances and BVs associated with host susceptibility and infectivity and thus cannot be ignored. The straight-forward approach of incorporating dynamic information in the form of incidence matrices into the mixed model framework using conventional animal breeding software is appealing due to ease of use and general availability and showed improvement over the standard IGE model. However this approach also has substantial limitations in incorporating disease dynamics. An alternative approach for incorporating epidemiological information and dynamic aspects would entail establishing an appropriate mathematical function that links the binary disease trait to underlying epidemiological parameters under genetic influence and encapsulates dynamic aspects.

## Conflict of Interest Statement

The authors declare that the research was conducted in the absence of any commercial or financial relationships that could be construed as a potential conflict of interest.
